# The role and intervention of mitochondrial metabolism in osteoarthritis

**DOI:** 10.1007/s11010-023-04818-9

**Published:** 2023-07-24

**Authors:** Zhanhai Qi, Jiaping Zhu, Wusheng Cai, Chunbiao Lou, Zongyu Li

**Affiliations:** 1 Department of Orthopedics, The 960th hospital of the Joint Logistics Support Force of the People’s Liberation Army, Jinan, Shandong China; 2 Department of Orthopedics, Jinan City People’s Hospital, Jinan, Shandong China; 3 Department of Orthopedics, Heze Third People’s Hospital, Heze, Shandong China

**Keywords:** Osteoarthritis, Mitochondria, Metabolism, Intervention

## Abstract

Osteoarthritis (OA), a prevalent degenerative joint disease, affects a substantial global population. Despite the elusive etiology of OA, recent investigations have implicated mitochondrial dysfunction as a significant factor in disease pathogenesis. Mitochondria, pivotal cellular organelles accountable for energy production, exert essential roles in cellular metabolism. Hence, mitochondrial dysfunction can exert broad-ranging effects on various cellular processes implicated in OA development. This comprehensive review aims to provide an overview of the metabolic alterations occurring in OA and elucidate the diverse mechanisms through which mitochondrial dysfunction can contribute to OA pathogenesis. These mechanisms encompass heightened oxidative stress and inflammation, perturbed chondrocyte metabolism, and compromised autophagy. Furthermore, this review will explore potential interventions targeting mitochondrial metabolism as means to impede or decelerate the progression of OA. In summary, this review offers a comprehensive understanding of the involvement of mitochondrial metabolism in OA and underscores prospective intervention strategies.

## Introduction

Osteoarthritis (OA) is a prevalent musculoskeletal disorder characterized by the degeneration of cartilage, subchondral bone, and other joint structures [1]. It represents a leading cause of pain and disability globally, particularly among the elderly population. The prevalence of OA rises with age, with approximately 10% of men and 18% of women aged 60 and above experiencing symptomatic OA worldwide [2]. This condition imposes a substantial burden on healthcare systems, resulting in high healthcare costs and productivity loss. In the United States alone, the annual cost of OA is estimated at $128 billion [3]. Risk factors for OA include advancing age, obesity, joint injury or trauma, genetic predisposition, and joint malalignment [4]. Unfortunately, there are currently no disease-modifying therapies available for OA, and the primary treatment goals revolve around pain relief and improvement of joint function. As a result, OA remains a significant unmet medical need and an area of active research.

**Fig. 1:**
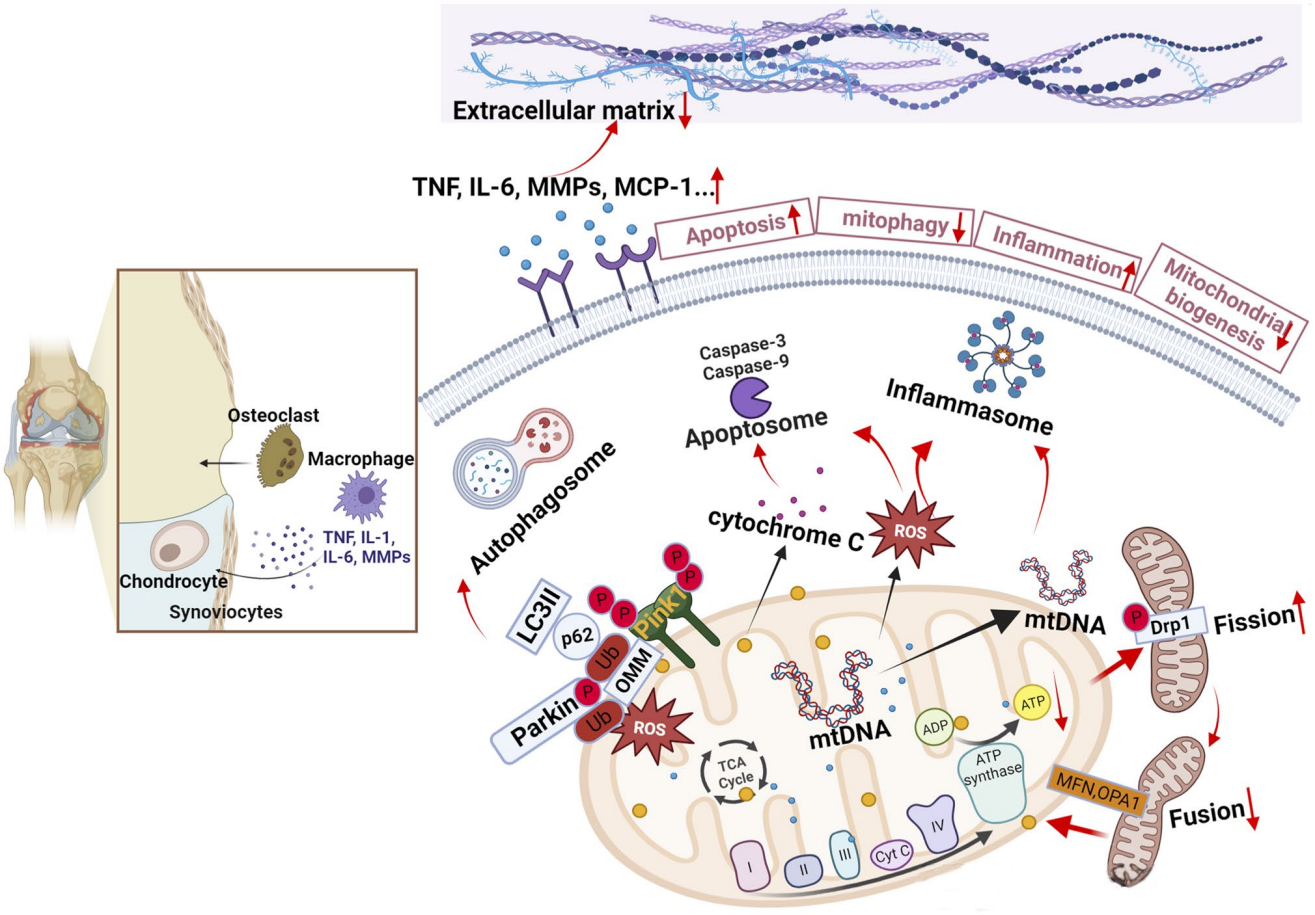
.

Mitochondria, the organelles responsible for cellular metabolism and energy production in eukaryotic cells, play a pivotal role in maintaining cellular homeostasis. They are the primary source of ATP, the universal energy currency, through a process called oxidative phosphorylation (OXPHOS) [5, 6]. In addition to energy generation, mitochondria contribute to various cellular processes, including apoptosis, calcium signaling, and the production of reactive oxygen species (ROS) [7]. Perturbations in mitochondrial function and metabolism have been implicated in numerous diseases, such as cancer, neurodegenerative disorders, and metabolic disturbances [8, 9]. Therefore, gaining a comprehensive understanding of the mechanisms underlying mitochondrial dysfunction is crucial for the development of effective therapeutic strategies for these conditions (Fig. 1).

Recent studies have revealed the significant contribution of mitochondrial dysfunction and disrupted energy metabolism to the pathogenesis of OA. Impaired mitochondrial function leads to the accumulation of ROS, which contribute to cartilage degradation and chondrocyte apoptosis. Research has demonstrated that elevated levels of mitochondrial ROS can accelerate cartilage degeneration and facilitate OA development [10]. Additionally, chondrocytes derived from OA patients exhibit reduced mitochondrial membrane potential and increased ROS production, resulting in oxidative stress and cell death [11]. Moreover, mitochondrial dysfunction impairs ATP production, leading to compromised chondrocyte function and cartilage degradation [12]. Animal studies have further substantiated the link between mitochondrial dysfunction and OA. For instance, an investigation utilizing a mouse model of OA demonstrated that inhibiting mitochondrial respiration promoted increased cartilage degradation and synovitis [13]. Notably, Durán-Sotuela et al. recently identified a correlation between the mtDNA variant m.16519C and an elevated risk of rapid knee OA progression [14]. Their findings indicated that m.16519C increased mtDNA copy number while decreasing mitochondrial biosynthesis. Furthermore, m.16519C led to heightened mitochondrial ROS levels, diminished expression of the mitochondrial fission-related gene fission mitochondrial 1, and impaired autophagic flux. These results further underscore the significant role of mitochondrial dysregulation in OA. Significantly, researchers have explored the potential involvement of mitochondrial biogenesis, the process responsible for the generation of new mitochondria, in OA. A study revealed that treatment with a mitochondrial biogenesis inducer resulted in enhanced mitochondrial function and improved cartilage health in a rat model of OA [15].

Collectively, these investigations highlight the involvement of mitochondrial dysfunction and altered metabolism in the development and progression of OA, thereby suggesting that targeting mitochondrial metabolism holds promise as a potential therapeutic strategy. However, further research is necessary to fully comprehend the underlying mechanisms of mitochondrial dysfunction in OA and to develop effective treatments targeting mitochondrial metabolism.

## Mitochondrial dysfunction in osteoarthritis

### Factors leading to mitochondrial dysfunction in osteoarthritis

Mitochondrial dysfunction has been implicated in the pathogenesis of OA. Studies indicate that mitochondrial dysfunction occurs prior to cartilage degradation and contributes to chondrocyte death [16]. Several factors have been identified as contributors to mitochondrial dysfunction in OA. Notably, oxidative stress is a key factor that can induce mtDNA damage, impair mitochondrial respiratory function, and activate mitochondrial-mediated cell death pathways [17]. Inflammatory cytokines and ROS produced by chondrocytes and synovial cells further exacerbate oxidative stress and mitochondrial dysfunction. Inflammatory cytokines such as IL-1β and TNF-α have been reported to reduce mitochondrial activity and ATP production, impair mitochondrial respiration in chondrocytes, and contribute to mitochondrial dysfunction in OA [18]. Additionally, proper mitochondrial dynamics, including fission and fusion, are critical for maintaining mitochondrial function. Increased fission and decreased fusion result in fragmented and dysfunctional mitochondria, leading to reduced ATP production and increased ROS generation [19]. Studies have shown that abnormal mitochondrial fission is associated with elevated phospho-Drp1 (Ser616) expression in OA chondrocytes [20]. Moreover, Zhang et al. reported downregulation of MFN1/2 and OPA1, along with abnormal translocation of DRP1 from the cytoplasm to the mitochondria, in OA chondrocytes [21]. Other mechanisms, such as altered mitochondrial biogenesis and mitophagy, have also been implicated in mitochondrial dysfunction in OA. Dysregulation of the PGC-1α/NRF-1 axis, a key regulator of mitochondrial biogenesis, has been observed in OA chondrocytes, leading to decreased mitochondrial mass and function [22]. Mitophagy, the selective degradation of damaged mitochondria, is disrupted in OA, resulting in the accumulation of dysfunctional mitochondria [10]. Abnormal expression of Parkin and P62, which mediate mitophagy, has been reported in OA [23]. Additionally, Kim et al. documented that the downregulation of PGC1α in OA could activate the PRKN-independent selective mitophagy pathway through the upregulation of BCL2 and BNIP3 [24]. In summary, multiple factors contribute to mitochondrial dysfunction in OA, including inflammation, oxidative stress, mitochondrial dynamics, biogenesis, and mitophagy. Understanding these mechanisms may offer potential targets for the development of novel therapies aimed at improving mitochondrial function and slowing or preventing the progression of OA.

### Changes in mitochondrial morphology and function in osteoarthritis

Mitochondria play a vital role in maintaining cellular function, and alterations in their morphology and function have been observed in OA. Studies utilizing human OA chondrocytes have demonstrated an increase in mitochondrial size and a decrease in mitochondrial number, indicating a shift towards elongated, dysfunctional mitochondria associated with oxidative stress and inflammation [25]. Recent literature suggests that the AMPK-SIRT3 positive feedback loop plays a critical role in regulating OA development and progression, partially by modulating chondrocyte mitochondrial quality [26]. Imbalances in mitochondrial fission and fusion may contribute to abnormal mitochondrial distribution and dysfunction, thereby influencing OA pathogenesis. Another investigation identified increased mitochondrial fragmentation in OA chondrocytes, resulting in reduced ATP production and heightened apoptosis [27]. It was reported that TBK1 participates in the OA process by directly phosphorylating DRP1 at Ser637, thereby influencing mitochondrial morphology remodeling [27]. In addition to morphological changes, mitochondrial function is also affected in OA, with decreased mitochondrial respiration and ATP synthesis observed in OA chondrocytes [23]. Furthermore, OA chondrocytes exhibit elevated mitochondrial oxidative stress and reduced antioxidant capacity, leading to mitochondrial DNA damage and dysfunction. Regulation of mitochondrial biogenesis is crucial for maintaining mitochondrial function, and evidence suggests a reduction in mtDNA content and a decrease in key regulators of mitochondrial biogenesis in OA, including PGC-1α and TFAM [28]. During OA progression, chondrocytes and synoviocytes tend to adapt their mitochondrial metabolism by shifting from oxidative phosphorylation to glycolysis, primarily regulated by the AMP-activated protein kinase (AMPK) and mechanistic target of rapamycin (mTOR) pathways [29]. Moreover, altered lipid and amino acid metabolism have been observed in these cells [29]. Additionally, changes in mitochondrial metabolism may lead to disturbances in cellular redox balance and the accumulation of reactive oxygen species (ROS) in OA. Recent research suggests that alterations in mitochondrial metabolism may contribute to the development of low-grade inflammation in OA [29]. Zhang et al. reported that Meta-Defensomes could reprogram the mitochondrial metabolism of M1 macrophages by scavenging mitochondrial ROS and inhibiting mitochondrial nitric oxide synthase, thereby increasing TFAM expression and restoring aerobic respiration, which suppresses synovial inflammation and early OA progression [30].

Overall, it is evident that mitochondrial dysfunction is a significant characteristic of OA and likely plays a key role in its development and progression. Therefore, gaining a better understanding of the mechanisms underlying mitochondrial dysfunction in OA holds promise for the development of new and effective therapeutic approaches to address this debilitating condition.

### The impact of mitochondrial dysfunction on chondrocytes and cartilage tissue

Studies have provided evidence of the substantial implications of mitochondrial dysfunction in chondrocytes for cartilage matrix production, chondrocyte apoptosis, and senescence, all of which contribute to the development and progression of OA [31]. Chondrocytes play a critical role in maintaining the extracellular matrix of cartilage tissue. Impaired mitochondrial function can disrupt ATP production, increase oxidative stress, and result in the accumulation of damaged proteins and lipids, all of which contribute to chondrocyte apoptosis [11]. Chondrocyte apoptosis is a key factor in cartilage degradation in OA, and mitochondrial dysfunction has been shown to heighten chondrocytes’ susceptibility to apoptosis triggered by mechanical stress, cytokines, and oxidative stress. The reduction in chondrocyte population due to apoptosis leads to diminished production of extracellular matrix components, including collagen and proteoglycans, ultimately resulting in cartilage degradation and the progression of OA [32].

Furthermore, mitochondrial dysfunction not only impacts chondrocyte apoptosis but also influences the composition and structure of the extracellular matrix in cartilage tissue. Studies have demonstrated that mitochondrial dysfunction can modulate the expression of matrix-degrading enzymes such as matrix metalloproteinases (MMPs), thereby contributing to extracellular matrix degradation [33]. Yang et al. reported that aurora kinase A (AURKA)-mediated degradation of SOD2 contributes to mitochondrial ROS and dysregulation of matrix metalloproteinase-13 (MMP-13), thus promoting the occurrence of OA through ubiquitination [34]. Additionally, mitochondrial dysfunction affects the production and organization of collagen and proteoglycans in cartilage tissue, leading to structural alterations and heightened vulnerability to mechanical stress [35]. Downregulation of GSK3β in chondrocytes enhances mitochondrial oxidative stress and damage, leading to increased nuclear translocation of Runx-2 and β-catenin, calcium deposition, cell death, and remodeling of the extracellular matrix, including MMP-1 and MMP-13 [35]. In summary, mitochondrial dysfunction exerts significant effects on chondrocytes and cartilage tissue, resulting in chondrocyte apoptosis, alterations in the composition of the extracellular matrix, and changes in bone metabolism. Understanding the underlying mechanisms of mitochondrial dysfunction in OA holds promise for the development of innovative therapies aimed at preventing and treating this debilitating disease.

## Mitochondrial metabolism and inflammation in osteoarthritis

Inflammatory processes play a pivotal role in the pathogenesis of OA, contributing to disease development and progression [36]. Key cytokines, including interleukin-1 (IL-1), tumor necrosis factor-alpha (TNF-α), and interleukin-6 (IL-6), have been identified as crucial drivers of inflammation in OA. These cytokines stimulate chondrocytes and synovial cells to produce matrix-degrading enzymes and pro-inflammatory mediators, resulting in cartilage degradation and joint destruction [37]. Besides cytokines, other factors such as adipokines, chemokines, and danger-associated molecular patterns (DAMPs) also contribute to the inflammatory response in OA. Adipokines, including adiponectin and leptin, produced by adipose tissue, induce the production of pro-inflammatory cytokines, thereby promoting inflammation [38]. Chemokines, such as monocyte chemoattractant protein-1 (MCP-1), recruit immune cells to the joint, further exacerbating inflammation [39]. DAMPs, such as high-mobility group box 1 (HMGB1) and S100A8/A9, activate toll-like receptors (TLRs) on immune cells, triggering an inflammatory response [40].

Targeting inflammation has been explored as a potential therapeutic strategy for OA. Nonsteroidal anti-inflammatory drugs (NSAIDs) are commonly employed to manage pain and inflammation in OA patients, but long-term use is associated with adverse effects such as gastrointestinal bleeding and cardiovascular events [41]. Other anti-inflammatory agents, including interleukin-1 receptor antagonists and tumor necrosis factor inhibitors, have been investigated for OA treatment, but their efficacy remains controversial [42, 43].

Recent studies have provided insights into the influence of mitochondrial dysfunction on modulating inflammatory processes in OA. Mitochondrial dysfunction can initiate the release of mitochondrial DNA (mtDNA) and mitochondrial reactive oxygen species (mtROS), activating the inflammasome and stimulating the production of pro-inflammatory cytokines such as interleukin-1β (IL-1β) and interleukin-18 (IL-18) in chondrocytes and synovial cells [44, 45]. These cytokines contribute to extracellular matrix breakdown and expedite OA progression. Additionally, mitochondrial dysfunction can impact the nuclear factor kappa B (NF-κB) signaling pathway, a key regulator of inflammation [46]. Mitochondrial ROS activate the NF-κB pathway, triggering the production of pro-inflammatory cytokines like TNF-α and IL-6 [47]. NF-κB activation also promotes the expression of matrix metalloproteinases (MMPs), contributing to extracellular matrix degradation and cartilage tissue damage [48].

Moreover, mitochondrial dysfunction can disturb the balance between pro-inflammatory and anti-inflammatory factors in synovial tissue. Accumulation of damaged mitochondria leads to the production of pro-inflammatory cytokines and chemokines such as monocyte chemoattractant protein-1 (MCP-1) and macrophage inflammatory protein-1α (MIP-1α) [49]. Furthermore, mitochondrial dysfunction decreases the production of anti-inflammatory factors like adiponectin, which impedes pro-inflammatory cytokine production and supports cartilage tissue repair [50]. Consequently, targeting mitochondrial dysfunction represents a promising therapeutic approach for managing inflammation in OA.

Several studies have proposed that targeting mitochondrial metabolism could serve as an effective therapeutic strategy for OA by addressing both mitochondrial dysfunction and inflammatory processes. Activation of the AMP-activated protein kinase (AMPK) pathway, known for its role in mitochondrial biogenesis and function, has demonstrated anti-inflammatory effects in OA chondrocytes [51]. Furthermore, the use of mitochondria-targeted antioxidants has been shown to reduce inflammation and protect against cartilage degradation in OA models [52]. Researchers have also investigated the therapeutic potential of targeting the mitochondrial pyruvate carrier (MPC), a critical regulator of mitochondrial metabolism, for various diseases [53]. Inhibiting MPC has been found to reduce mitochondrial respiration, ROS production, and the production of inflammatory mediators [54], making it a potential therapeutic approach for OA. Additionally, activation of hypoxia-inducible factor 1-alpha (HIF-1α) has been shown to decrease inflammatory cytokine synthesis, preserve the chondrogenic phenotype, regulate glycolysis and mitochondrial function in OA, and delay cartilage degradation by promoting a denser collagen matrix [55]. Hence, HIF-1α represents a crucial therapeutic target for OA by regulating chondrocyte inflammation and mitochondrial metabolism. Moreover, blocking LncHOTAIR has been found to improve mitochondrial activity, suppress IL-1β-induced chondrocyte inflammation, and reduce ROS levels in OA via the miR-222-3p/ADAM10 axis, suggesting LncHOTAIR as a potential therapeutic target for OA [56].

Furthermore, recent studies have indicated that the gut microbiota plays a role in regulating mitochondrial metabolism and inflammation in OA. Imbalances in the gut microbiota, referred to as dysbiosis, have been associated with increased inflammation and mitochondrial dysfunction in OA models [57]. Treatment with probiotics or prebiotics has been shown to restore gut microbial balance, improve mitochondrial function, decrease inflammation, and mitigate cartilage degradation [57]. Another potential drug target is peroxisome proliferator-activated receptor gamma coactivator 1-alpha (PGC-1α), a transcriptional coactivator involved in mitochondrial biogenesis and function. Activation of PGC-1α has demonstrated the ability to enhance mitochondrial function and reduce inflammation in chondrocytes and animal models of OA [58].

Mitochondrial dysfunction and inflammation are closely interconnected in the development and progression of OA. Targeting mitochondrial metabolism holds promise as a therapeutic strategy for OA, with natural compounds and pharmacological agents aimed at improving mitochondrial function and reducing inflammation showing potential. However, further research is necessary to establish optimal dosing and treatment durations, evaluate potential side effects, and explore the potential of gene therapy approaches.

## Mitochondrial metabolism and apoptosis in osteoarthritis

Mitochondria plays a pivotal role in the regulation of apoptosis, and their dysfunction can initiate apoptotic pathways. Apoptosis is a tightly controlled process that can be triggered through intrinsic and extrinsic pathways. In patients with OA, mitochondrial dysfunction has been associated with the activation of caspase-3, a key mediator of chondrocyte apoptosis [59]. Imbalances in apoptosis regulation have been observed in chondrocytes of OA patients, characterized by increased expression of the pro-apoptotic protein Bax and decreased expression of the anti-apoptotic protein Bcl-2 [60]. These imbalances are correlated with reduced mitochondrial membrane potential and elevated production of mitochondrial ROS, both indicative of mitochondrial dysfunction [60].

Moreover, mitochondrial damage can lead to the release of cytochrome c from the mitochondrial intermembrane space into the cytosol, where it activates the caspase-9 pathway and subsequently triggers caspase-3 activation [61]. Additionally, mitochondrial dysfunction can influence autophagy, a cellular process responsible for the degradation of damaged organelles and proteins, ultimately leading to apoptosis [62]. Disrupted mitochondrial metabolism can activate cell death pathways while inhibiting cell survival pathways, contributing to various diseases. In the context of OA, mitochondrial dysfunction contributes to chondrocyte apoptosis and cartilage degeneration. Studies have reported that Regulated in Development and DNA Damage Response 1 (REDD1) downregulates mitochondrial biogenesis markers, such as PGC-1α and TFAM, leading to chondrocyte death in a mouse model of OA [63]. Furthermore, orphan nuclear receptor subfamily 4 group A member 1 (NR4A1), an important transcription factor, promotes mitochondrial dysfunction and triggers chondrocyte apoptosis in OA [64]. Dysregulation of mitochondrial dynamics markers, including Drp1 (a mitochondrial fission marker), Tom20 (a mitochondrial outer membrane protein), and MFN1 (a mitochondrial fusion marker), has been associated with IL-1β-induced chondrocyte apoptosis, suggesting that IL-1β-induced mitochondrial dynamics dysfunction may accelerate chondrocyte apoptosis [11, 65].

Recent research has explored the potential of mitochondria-targeted therapies to prevent chondrocyte apoptosis in OA. One approach involves the use of mitochondrial-targeted antioxidants, which accumulate within mitochondria to scavenge ROS produced by dysfunctional mitochondria, thus mitigating chondrocyte apoptosis. Delco et al. demonstrated that treatment with the mitochondrial-targeted antioxidant SS-31 reduced ROS levels and prevented cartilage degradation in a rat model of OA [66]. Similarly, Liu et al. showed that treatment with the mitochondrial division inhibitor Mdivi-1 reduced chondrocyte apoptosis and cartilage degradation in a rat model of OA [67]. Additionally, Verhagen et al. found that treatment with the mitochondrial permeability transition pore inhibitor cyclosporin A reduced chondrocyte apoptosis and cartilage degradation in a rat model of OA [68]. Synthetic mitochondrial-targeted antioxidants, such as MitoQ and SkQ, have been investigated in various diseases to decrease mitochondrial ROS production, inhibit cell inflammation and apoptosis, and hold promise for OA treatment [69, 70]. Another strategy involves the use of small molecules that specifically target mitochondrial pathways involved in apoptosis, such as the Bcl-2 family proteins. For example, ABT-263 can inhibit the anti-apoptotic protein Bcl-2 and induce chondrocyte apoptosis in OA [71]. Additionally, circFAM160A2 has been reported to promote mitochondrial stabilization and suppress apoptosis in OA chondrocytes by targeting miR-505-3p and SIRT3, offering a potential therapeutic target for OA therapy [72]. Furthermore, AMPK activation via SIRT3 has been shown to limit oxidative stress, suppress apoptosis, and improve mitochondrial DNA integrity and function in OA chondrocytes, highlighting the protective effect of AMPK-SIRT3 activation in OA [73]. Therefore, targeting mitochondrial metabolism and function emerges as a promising therapeutic strategy for managing OA.

## Mitochondrial metabolism and cartilage matrix degradation in osteoarthritis

Cartilage matrix degradation is a characteristic feature of OA, and mounting evidence suggests that mitochondrial dysfunction contributes to this process. Several mechanisms have been identified through which mitochondrial dysfunction influences the degradation of the extracellular matrix (ECM) in cartilage. One mechanism involves the activation of MMPs, which are responsible for ECM degradation [74]. Mitochondrial dysfunction can upregulate MMP expression and activity in chondrocytes, leading to the breakdown of ECM components such as collagen and aggrecan [75]. Inhibition of mitochondrial dysfunction using various compounds has been shown to reduce MMP activity and attenuate cartilage degradation in OA models [76, 77]. Furthermore, oxidative stress resulting from mitochondrial dysfunction can contribute to cartilage matrix degradation. ROS generated during mitochondrial dysfunction can oxidize and cleave ECM components, thereby promoting matrix degradation [78].

The impact of mitochondrial dysfunction on matrix-degrading enzymes in chondrocytes has also been documented. It has been observed that mitochondrial dysfunction increases the production of MMPs, which play a pivotal role in ECM degradation, including collagen and proteoglycans [79]. Mitochondrial dysfunction activates signaling pathways such as MAPKs and NF-κB, leading to the upregulation of MMP expression and activity in chondrocytes. For example, inhibition of mitochondrial respiratory chain complex III has been found to upregulate MMP expression in human chondrocytes [80]. Hydrogen peroxide-induced mitochondrial dysfunction has also been shown to increase MMP-13 expression and activity in chondrocytes [81]. Moreover, mitochondrial dysfunction has been linked to alterations in the expression and activity of other matrix-degrading enzymes, such as ADAMTS and cathepsins. Studies have revealed that mitochondrial dysfunction upregulates the expression and activity of ADAMTS-5, which contributes to aggrecan degradation [82]. Additionally, mitochondrial dysfunction has been associated with increased expression and activity of cathepsins B, which are involved in the degradation of type II collagen, another vital component of cartilage ECM [83]. These findings emphasize the important role of mitochondrial dysfunction in regulating matrix-degrading enzymes in chondrocytes, thereby contributing to cartilage matrix degradation in OA.

Recent evidence suggests that targeted therapies aimed at mitochondria may hold promise in reducing cartilage matrix degradation and halting the progression of OA. Several studies have demonstrated that inhibition of mitochondrial complex I or II can diminish cartilage matrix degradation and enhance chondrocyte survival in animal models of OA [84]. Furthermore, promoting mitochondrial fusion and inhibiting fission has been shown to improve mitochondrial function and reduce cartilage matrix degradation [27]. Compounds capable of modulating mitochondrial metabolism and improving mitochondrial function, such as nicotinamide riboside and pyrroloquinoline quinone, have also been investigated in the context of OA [85, 86]. These compounds promote mitochondrial biogenesis, enhance mitochondrial respiration, and reduce oxidative stress and inflammation in chondrocytes, ultimately preserving the integrity of the cartilage matrix. Additionally, Hung et al. reported that inhibiting the SIRT1/AMPK/PGC-1α signaling pathway in chondrocytes resulted in mitochondrial dysfunction characterized by increased oxidative stress and apoptosis, leading to cartilage matrix loss through upregulation of MMP-13 expression. This finding provides a theoretical basis for understanding OA etiology and intervention [87]. Furthermore, inhibition of LncHOTAIR has been found to improve mitochondrial activity and mitigate cartilage matrix degradation by regulating MMP-13, suggesting its potential role in OA intervention [56].

## Mitochondrial metabolism and autophagy in osteoarthritis

Autophagy, a critical cellular process involved in maintaining cellular homeostasis, is responsible for the degradation of unwanted or damaged organelles and proteins through lysosomal degradation pathways. This process entails the formation of autophagosomes, double-membraned vesicles that sequester cytoplasmic cargo and subsequently fuse with lysosomes to form autolysosomes for degradation [88]. In the context of chondrocytes and cartilage homeostasis, autophagy plays a vital role [89]. It facilitates the degradation of misfolded proteins and damaged organelles, including mitochondria, thereby maintaining a healthy chondrocyte phenotype [89]. Moreover, autophagy is involved in regulating chondrocyte apoptosis and cartilage matrix degradation in OA [90]. Studies have demonstrated that inhibiting autophagy in an OA mouse model leads to increased cartilage damage and chondrocyte apoptosis, while activating autophagy promotes chondrocyte survival and reduces matrix degradation in OA [11, 91]. However, it is worth noting that while autophagy serves as a stress adaptation mechanism to prevent cell death, excessive autophagy can also lead to cellular demise [92]. Mitochondrial dysfunction plays a significant role in disrupting autophagy and contributing to the development of various diseases, including OA. Research has shown that mitochondrial dysfunction in OA chondrocytes impairs autophagy, exacerbating mitochondrial damage and oxidative stress within the cells [93]. Impaired autophagy is associated with the accumulation of damaged mitochondria and the activation of inflammatory pathways, both of which contribute to the pathogenesis and progression of OA [91]. Kim et al. reported that mitochondrial dysfunction reduces autophagy activity in chondrocytes, resulting in increased apoptosis and cartilage degeneration [24].

Enhancing autophagy represents a potential therapeutic strategy for treating OA, and targeting mitochondrial dysfunction and improving mitochondrial health can play a crucial role in regulating autophagy and promoting chondrocyte survival. Studies have shown that administration of mitochondrial-targeted antioxidants, such as MitoQ and MitoTEMPO, can restore autophagy and reduce cartilage degeneration [60, 94]. Wang et al. demonstrated that metformin can regulate the mitophagy process through the SIRT3-PINK1-PRKN signaling pathway, counteracting oxidative stress and imbalance of anabolism and catabolism induced by IL1B in chondrocytes, thus highlighting metformin’s potential in the prevention and treatment of OA through modulation of mitophagy [95]. Additionally, zinc has been found to reverse disturbances in mitochondrial metabolism and mitophagy induced by monosodium iodoacetate, suggesting its potential protective role against OA progression [84]. Overexpression of circErcc2 has also been shown to attenuate apoptosis, ECM degradation, and enhance mitophagy by targeting Mir182-5p-SIRT1 in response to oxidative stress, offering potential therapeutic approaches for OA [10]. In summary, these findings support the notion that mitochondrial-targeted therapies hold promise in regulating autophagy and promoting chondrocyte survival in OA.

## Conclusion and future direction

In summary, the role of mitochondrial metabolism in the development of OA is crucial. Dysfunctional mitochondria in chondrocytes contribute to the generation of ROS, oxidative stress, inflammation, and apoptosis [96]. Moreover, impaired cellular energetics and autophagy contribute to extracellular matrix degradation, resulting in cartilage loss and OA progression [11]. Mitochondrial-targeted therapies, including antioxidants, mitochondrial biogenesis activators, and mitophagy modulators, hold promise in mitigating mitochondrial dysfunction and associated pathological changes in OA. Currently, no approved drugs specifically targeting mitochondrial metabolism in OA exist. However, preclinical and clinical studies have explored various treatments with potential efficacy in this regard. For example, metformin, a widely used antidiabetic medication, activates AMPK and has been shown to ameliorate mitochondrial dysfunction and protect against cartilage damage in OA models [95, 97]. Clinical trials are underway to assess the potential benefits of metformin in human OA patients. Additionally, specific nutraceuticals and dietary supplements have been investigated for their effects on mitochondrial metabolism in OA. Coenzyme Q10 (CoQ10), an essential component of the mitochondrial electron transport chain, has shown promise in improving mitochondrial function and reducing pain in OA patients [98, 99]. Likewise, resveratrol, a polyphenol found in grapes and berries, has been studied for its antioxidant and anti-inflammatory properties, which may contribute to enhanced mitochondrial function in OA [100, 101]. A clinical trial demonstrated that hyaluronic acid treatment in OA had a significantly superior effect to methylprednisolone, partially attributed to improved mitochondrial function [102]. Nonetheless, further research is necessary to fully comprehend the intricate interactions between mitochondrial metabolism and OA pathophysiology. The development and optimization of mitochondrial-targeted therapies hold the potential to provide effective prevention and treatment strategies for this prevalent and debilitating joint disease.

Although mitochondrial-targeted therapies have demonstrated promise in preclinical investigations as a potential treatment for OA, further research is required to comprehensively assess their efficacy, safety, and long-term effects in human subjects. Subsequent studies could focus on optimizing the delivery and dosage regimens of these therapies, as well as investigating potential synergistic effects when combined with existing treatments such as nonsteroidal anti-inflammatory drugs and corticosteroids. Additionally, more investigations are warranted to elucidate the molecular mechanisms underlying the impact of mitochondrial dysfunction in OA and to identify novel therapeutic targets. Recent advancements in high-throughput screening technologies and genomics have facilitated the identification of new drug candidates capable of modulating mitochondrial metabolism and function, offering potential avenues for the development of innovative OA treatments. Another area of interest pertains to the development of biomarkers that could be utilized to monitor the effectiveness of mitochondrial-targeted therapies in OA patients. Overall, mitochondrial metabolism assumes a critical role in the pathogenesis of OA, and targeting mitochondrial dysfunction holds promise as a prospective therapeutic strategy for addressing this incapacitating condition.

At present, preclinical studies have shown potential benefits associated with mitochondrial-targeted antioxidants and other therapies. Nonetheless, further research is necessary to ascertain optimal dosages, administration routes, and potential adverse effects in humans. If successful, mitochondrial-targeted therapies may provide disease-modifying treatment options for OA by promoting chondrocyte survival and reducing degradation of the cartilage matrix, thereby affording long-term benefits. Furthermore, these therapies have the potential to diminish the reliance on conventional pain management strategies, including nonsteroidal anti-inflammatory drugs and opioids, which carry significant side effects and risks.

It should be noted that mitochondrial-targeted therapies are not meant to be standalone solutions and can be employed in conjunction with existing treatments for OA. The integration of mitochondrial-targeted therapies within a multimodal approach to OA treatment may yield improved outcomes for patients.

## Data Availability

Enquiries about data availability should be directed to the authors.
